# The Role of Toll-Like Receptor-2 in *Clostridioides difficile* Infection: Evidence From a Mouse Model and Clinical Patients

**DOI:** 10.3389/fimmu.2021.691039

**Published:** 2021-07-12

**Authors:** Yi-Hsin Lai, Bo-Yang Tsai, Chih-Yu Hsu, Yi-Hsuan Chen, Po-Han Chou, Yueh-Lin Chen, Hsiao-Chieh Liu, Wen-Chien Ko, Pei-Jane Tsai, Yuan-Pin Hung

**Affiliations:** ^1^ Institute of Basic Medical Sciences, College of Medicine, National Cheng Kung University, Tainan, Taiwan; ^2^ Department of Medical Laboratory Science and Biotechnology, National Cheng Kung University, Tainan, Taiwan; ^3^ Department of Internal Medicine, National Cheng Kung University Hospital, Tainan, Taiwan; ^4^ Departments of Internal Medicine, Tainan Hospital, Ministry of Health & Welfare, Tainan, Taiwan; ^5^ Department of Medicine, College of Medicine, National Cheng Kung University, Tainan City, Taiwan; ^6^ Center of Infectious Disease and Signaling Research, National Cheng Kung University, Tainan City, Taiwan; ^7^ Department of Pathology, National Cheng Kung University Hospital, Tainan, Taiwan

**Keywords:** *Clostridioides difficile* infection, toll-like receptor, TLR2, rs3804099, tight junction, mice model

## Abstract

**Background:**

*Clostridioides difficile* is the leading cause of nosocomial infectious diarrhea. Toll-like receptors (TLRs) are the major components of innate immunity that sense pathogens. The relationship between TLRs and *C. difficile* infection (CDI) was analyzed in clinical patients and a mouse model.

**Materials and Methods:**

A prospective investigation was conducted in medical wards of Tainan Hospital, Ministry of Health and Welfare, Tainan, Taiwan, from January 2011 to January 2013. Adult patients were followed up for the development of CDI. Single nucleotide polymorphisms (SNPs) of TLR2 and TLR4 were analyzed to assess the relationship between genetic polymorphisms and the development of CDI. A mouse model of CDI was used to investigate the pathogenic role of TLRs in CDI, TLR2 and TLR4 knockout (Tlr2-/- and Tlr4-/-) mice.

**Results:**

In the prospective study, 556 patients were enrolled, and 6.5% (36) of patients, accounting for 3.59 episodes per 1000 patient-days, developed CDI. Of 539 patients with available blood samples, the TLR2 rs3804099 polymorphism was more often noted in those with CDI than in those without CDI (64.5% *vs.* 46.1%; *P* = 0.046) but was not significant in multivariate analysis. Because the TLR2 rs3804099 polymorphism was moderately associated with CDI, the role of TLR2 and TLR4 was further evaluated in a mouse model. Both Tlr2-/- and Tlr4-/- mice showed more severe CDI disease than wild-type mice in terms of body weight change and fecal content five days after oral challenge with *C. difficile*. Furthermore, Tlr2-/- mice suffered from more severe disease than Tlr4-/- mice, as evidenced by stool consistency, cecum weight, and survival rate.

**Conclusion:**

The TLR2 rs3804099 polymorphism is marginally associated with the development of CDI, and the pathogenic role of TLR2 is further supported by a mouse model.

## Introduction

*Clostridioides difficile* is the leading cause of nosocomial infectious diarrhea, with clinical symptoms ranging from mild diarrhea to pseudomembranous colitis and toxic megacolon (1–7). Increased virulence of the *C. difficile* NAP-1/027 epidemic clone in Quebec is known to be associated with increased production of toxins A and B, fluoroquinolone resistance, and production of binary toxins (8). Although the pathogenesis of *C. difficile* was largely attributed to the production of toxins A and B by bacteria, vaccination with the toxins was unable to fully protect animals from *C. difficile* infection (CDI) (9). Clinical trials of actoxumab and bezlotoxumab, human monoclonal antibodies against *C. difficile* toxins A and B, respectively, revealed that the initial clinical cure rates were 80% with bezlotoxumab alone, 73% with actoxumab plus bezlotoxumab, and 80% with placebo; the rates of sustained cure (initial clinical cure without recurrent infection in 12 weeks) were 64%, 58%, and 54%, respectively (10). The inability to fully protect against CDI by toxin-specific monoclonal antibodies suggests that other factors may contribute to disease progression. Impaired host immunity, recent receipt of antibiotics, anticancer chemotherapy, proton-pump inhibitors or H2 blockers, the presence of comorbidities with functional impairment, immune gene polymorphisms (such as CXCL8), and low levels of antibodies against *C. difficile* toxin B were identified as risk factors for CDI and disease recurrence (8, 11, 12). However, the major component of protective immunity against CDI remains undefined.

Toll-like receptors (TLRs) are a class of single membrane-spanning receptors and play a role in recognizing invading organisms by innate immune cells (13, 14). The most widely investigated TLRs in the pathophysiology of CDI are TLR5 (15–17) and TLR9 (18). Through the TLR5 pathway, *C. difficile* flagellin could induce the activation of NF-kappaB (which is downstream of TLRs) and p38 mitogen-activated protein kinase and promote the production of interleukin-8 and CCL20 (16). The TLR5 pathway induced by purified Salmonella-derived flagellin, a TLR5 agonist, protected mice from CDI by delaying *C. difficile* growth and toxin production in the colon and cecum (17). TLR5 pathway activation prompted by a recombinant fusion protein vaccine containing the nontoxic domains of *C. difficile* toxins A and B provided immunogenicity and protective efficacy in a mouse model of CDI (15). TLR9 is activated during CDI when TcdA and TcdA fragments remodel membranes of human colonocytes, monocytes and macrophages, which allows them to access endosomes and present bacterial DNA (18).

TLR2 and TLR4, two other important TLRs in innate immunity, have been noted to play an important role in many gram-positive or gram-negative bacterial infections. Single nucleotide polymorphisms (SNPs) refer to single nucleotide differences in some DNA sequences in the homologous interval (19). SNPs in TLR might affect receptor function through their effects on TLR expression, localization, trafficking and signaling (19). Patients with SNPs of TLR2 have been shown to be more susceptible to gram-positive infections, such as *Bacillus subtilis, Staphylococcus aureus, Streptococcus pneumoniae*, and *Listeria monocytogenes*, by identifying components of bacterial cell walls (20, 21). In contrast, patients with certain TLR4 polymorphisms are predisposed to intestinal infections by gram-negative organisms, Crohn’s disease, or ulcerative colitis based on the detection of lipopolysaccharide (LPS) (20, 21).

The role of TLR4 in the pathophysiology of CDI has been investigated in recent years (22). In a mouse model, surface layer proteins of *C. difficile* can activate innate and adaptive immunity by the TLR4-mediated signaling pathway (22). In our prospective clinical study, the TLR4 rs1927914 polymorphism (GG genotype) was associated with *C. difficile* colonization, suggesting that TLR4 builds adaptive immunity against the existence of *C. difficile* in the gut (23). The role of TLR2 and TLR4 in host immunity against CDI needs further study. The correlation between TLR2 and TLR4 polymorphisms and the occurrence of CDI in adult patients and the pathogenic role of TLR2 and TLR4 in an established mouse model of CDI were investigated in this study.

## Materials and Methods

### Hospitalized Patients

A prospective study was conducted in the medical wards of Tainan Hospital, Ministry of Health and Welfare, a district hospital in southern Taiwan, from January 2011 to January 2013. The study was approved by the institutional review board of Tainan Hospital, Ministry of Health and Welfare, Taiwan (approval number: IRB-2011014), and written informed consent was obtained from all patients. The inclusion criteria included both males and females aged at least 20 years admitted to medical wards. Patients with CDI within the previous three months, metronidazole or oral vancomycin therapy within the previous three months, colectomy, CDI at admission, or gastrointestinal infections due to identified enteropathogens were excluded.

Information about clinical status prior to admission, including previous CDI or medication, was prospectively queried. Clinical data, including age, nasogastric tube use or underlying disease, were recorded. SNPs of TLR2 and TLR4 were selected for analysis in our study according to two criteria: first, the SNPs had been reported to have a high frequency (minor allele frequency more than 0.05) in the Chinese population, and second, the SNPs had been reported to be associated with inflammatory or immunological responses. Probe sequences used for detecting TLR2 and TLR4 polymorphisms are shown in **Supplementary Table 1**. Three SNPs, rs1898830 (-15,607A/G, located at Intron 1, chr4:153687301 (GRCh38.p12), NC_000004.12:g.153687301A>G), rs3804099 (located at Exon 3, 4:153703504 (GRCh38), NC_000004.12:153703503:T:C), and rs7656411 (located at 3’-flanking, 4:153706503 (GRCh38), NC_000004.12:153706502: T:C,NC_000004.12: 153706502:T:G) of the TLR2 gene, were characterized as high-frequency SNPs in the Chinese cohort, and two of them (rs1898830 and rs3804099) were significantly associated with cytokine production by peripheral blood leukocytes in response to bacterial lipoprotein stimulation (24). Two alleles, rs10983755 (9:117702392 (GRCh38), NC_000009.12:117702391:G:A) and rs1927914 (9:117702447 (GRCh38), NC_000009.12:117702446:G:A), are located in the 5’ flanking region of the *TLR4* gene (25); these polymorphisms of the 5′ flanking region may have functional consequences for TLR4 expression or signaling activity (26, 27) and are highly prevalent in the Chinese population (25, 28).

Since each chromosome consisted of two alleles, the results indicated the SNPs, taking rs1898830 as an example, might be homozygotes (such as AA and GG genotypes) or heterozygotes (GA genotypes). In our study, one homozygote [for example, the AA genotype, selected according to a reference article (24)] was compared to other genotypes (the GA+ GG genotype, or collectively named G-carrier) to analyze the correlation between different genotypes and the occurrence of CDI.

Sampling procedures and probe sequencing have been described previously (23). If a hospitalized patient developed diarrhea, a stool sample was sent for cultures within less than one hour of collection, plated on cycloserine-cefoxitin-fructose agar (CCFA), and cultured under anaerobic conditions. Diarrhea was defined as a change in bowel habit with more than three unformed bowel movements per day for at least two days. Those with fecal *C. difficile* isolates possessing *tcdB* in the presence of diarrhea without an alternative explanation were diagnosed as having CDI (11), which was the primary outcome of the clinical study. Multiplex PCR was used to detect *tcdA*, *tcdB*, *cdtA*, and *cdtB* and a *tcdC* deletion as previously described (29). For patients with CDI, the period from admission to the first episode of CDI, or for those without CDI, the period from admission to discharge, was recorded.

Statistical analysis was performed by statistical software (SPSS, version 13.0). Continuous data are expressed as the means ± standard deviations. The χ^2^ test or Fisher’s test was used for categorical variables, and Student’s *t*-test was used for continuous variables. A two-tailed *P* value of less than 0.05 was considered to be statistically significant. The parameters with *P* values less than 0.05 in the univariate analysis were entered into a multivariate analysis with a binary logistic regression model. The Bonferroni correction for multiple testing was applied.

### CDI Mouse Model

A toxigenic *C. difficile* strain, VPI 10463 (CCUG 19126 or ATCC 43255), without binary toxin was used. To disrupt the intestinal microbiota, the mice were given drinking water containing an antibiotic mixture, which included 0.045 mg/mL vancomycin, 0.215 mg/mL metronidazole, 0.4 mg/mL kanamycin, 0.035 mg/mL gentamicin, and 850 U/ml colistin, from 5 days to 1 day before oral inoculation of vegetative *C. difficile* bacteria. The mice received a PPI, esomeprazole (40 mg/kg/day) or phosphate-buffered saline (PBS) twice daily for 2 days before oral inoculation of *C. difficile* as we had reported (30). Then, 3.5 x 10^7^ CFUs of *C. difficile* VPI10463 vegetative cells were administered orogastrically, and clindamycin was intraperitoneally injected at a dose of 4 mg/kg. Luciferin (Xenogen, USA), a luciferase substrate, was intraperitoneally injected at a dose of 150 mg/kg (30 mg/ml) before imaging to demonstrate NF-κB activation-mediated luminescence.

### Phenotypic Evaluation of CDI

Reported signs of colitis in mice included weight loss and diarrhea (31). Diarrhea was scored by stool consistency, as follows: 0 = well-formed pellets, 1 = semiformed stools that did not adhere to the anus, 2 = semiformed stools that adhered to the anus, and 3 = liquid stools. Thus, changes in body weight, stool consistency, gross view of the gut, and cecal weight were selected to estimate the disease severity of CDI in mice.

#### *C. difficile* Mouse Model Pathologic Scoring System

For pathological scoring, six fields per sample were examined and scored. Average counts of neutrophils and eosinophils in the six high-power fields (HPFs) in tissues were examined. The severity of colitis was scored, ranging between zero and three points for each of the following parameters: (i) polymorphonuclear infiltrate; (ii) mononuclear infiltrate; (iii) edema; (iv) erosion and ulcerations; (v) crypt abscess; (vi) crypt destruction; and (vii) distribution of inflammation (mucosa =1, mucosa and submucosa =2, transmural inflammation =3). Based on the total score (range from 0 to 21), inflammation was graded as mild (1 ± 5 points), moderate (6 ± 10 points) or severe (>10 points) (32).

### Toll-Like Receptor Knockout (KO) Mice

Mice deficient in TLR2 (Tlr2-/-) and TLR4 (Tlr4-/-) were purchased from NCKU Laboratory Animal Center (transferred from National Laboratory Animal Center), and the National Laboratory Animal Center in Tainan maintained the mice on a C57BL/6 background. The control group was age-matched wild-type C57BL/6 mice obtained from the National Laboratory Animal Center, Taiwan. All mice were housed in a specific pathogen-free facility at NCKU Animal Center under a light-dark cycle of 12-12 h with humidity and temperature control and provided food and water. All of the mouse studies, including the survival rate experiments, with six mice in each group were repeated more than three times to confirm the results.

### RNA Analysis

Tissue RNA was extracted by TRIzol reagent (Invitrogen, Carlsbad, California), and mRNA levels were analyzed with real-time quantitative RT-PCR (Applied Biosystems, Foster City, California) using β-actin or GAPDH as reference genes in each reaction.

### Protein Analysis

The protein extracts were separated by SDS-PAGE and transferred to PVDF membranes (Pall Gelman Laboratory, Ann Arbor, MI). After blocking the membrane, protein extracts were incubated with rabbit antibodies against TLR2 (Abcam). The membranes were then incubated with horseradish peroxidase-conjugated goat anti-rabbit IgG (Calbiochem), and the antigens were detected by an enhanced chemiluminescence Western blotting detection system (Amersham Life Science Increase).

## Results

### Clinical Cases

Of 539 patients with available blood samples, 5.8% (31) of patients, accounting for 3.49 episodes/1000 patient-days, developed CDI in the hospital. Patients with CDI more frequently had diabetes mellitus (64.5%, *vs.* 32.7%; *P* < 0.001) and prior exposure to PPIs than those without CDI (30.6% *vs.* 15.2%; *P* = 0.02, **Supplementary Table 2**). The TT genotype of the TLR2 rs3804099 polymorphism was more often noted in those with CDI than in those without CDI (64.5% *vs.* 46.1%; *P* = 0.046), but no correlation was present between other TLR2 (rs1898830 and rs7656411) or TLR4 (rs10983755 and rs1927914) polymorphisms and CDI (**Table 1**). In the multivariate analysis, diabetes mellitus (OR 3.61, 95% CI 1.66-7.84, *P* = 0.001), prior cephalosporin (OR 10.48, 95% CI 1.40-78.33, *P* = 0.02) and PPI therapy (OR 3.02, 95% CI 1.35-6.79. P = 0.007) were independently related to CDI (**Table 2**). The TLR2 rs3804099 polymorphism was correlated with CDI but without statistical significance [odds ratio (OR) 2.06, 95% confidence interval (CI) 0.94-4.50, *P* = 0.07]. Because the TLR2 rs3804099 polymorphism was doubtfully associated with CDI, the role of TLR2 and TLR4 in CDI was further evaluated in the mouse model.

**Table 1:** Correlation of TLR-2 or TLR-4 polymorphisms and the development of *Clostridioides difficile* infection (CDI) in 539 patients.

Polymorphism	CDI		*P* value
	No, n = 508	Yes, n = 31	
TLR-2			
rs1898830 (n=445)			0.19
AA genotype	140 (33.0)	3 (14.3)	
GA+GG genotype	284 (67.0)	18 (85.7)	
rs3804099 (n=539)			0.046
TT genotype	234 (46.1)	20 (64.5)	
TC+CC	274 (53.9)	11 (35.5)	
rs7656411 (n=455)			0.85
TT genotype	109 (25.7)	5 (23.8)	
TG+GG genotype	315 (74.3)	16 (76.2)	
TLR-4			
rs 10983755 (n=539)			0.71
AA genotype	30 (5.9)	2 (6.5)	
AG+GG genotype	478 (94.1)	29 (93.5)	
rs1927914 (n=539)			0.85
AA genotype	196 (38.6)	11 (35.5)	
AG+GG genotype	312 (61.4)	20 (64.5)	

Data are no. (%) of patients, unless otherwise indicated.

**Table 2:** Multivariate analysis of risk factors for CDI in 539 patients.

Characters	Odds ratio	95% confidence interval	*P* value
TLR2 rs3804099 polymorphism, TT type	2.06	0.94-4.50	0.07
Prior proton pump inhibitor therapy	3.02	1.35-6.79	0.007
Diabetes mellitus	3.61	1.66-7.84	0.001
Prior cephalosporin therapy	10.48	1.40-78.33	0.02

### Mouse Model of CDAD

#### Tissue Expression of TLR2 and TLR4 in Mice With CDI

**Figure 1:**
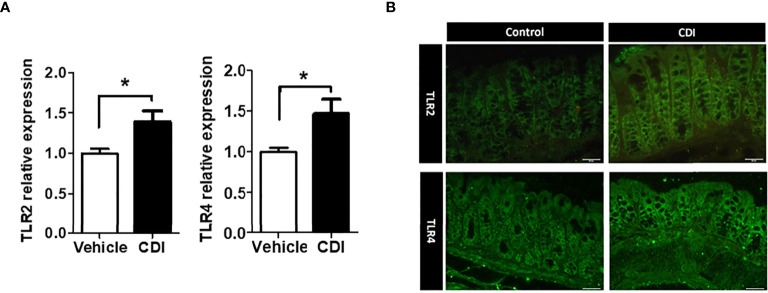
The expression of TLR2 and TLR4 increased in wild-type mice with CDI compared with uninfected mice (vehicle) in protein expression **(A)** and in IHC staining **(B)**. Values are expressed as means ± SEMs (*P < 0.05).

RNA expression levels and immunohistochemistry (IHC) staining were used to examine TLR2/4 protein expression and localization in colon and rectum specimens in a mouse model. The results showed a 40% increase in TLR2 protein expression and a 48% increase in TLR4 protein expression in the colon and rectum of *C. difficile*-infected mice (**Figure 1**). Increased expression of TLR2 and TLR4 was also noted by IHC staining (**Figure 1**).

#### Increased Disease Severity in TLR2-/- or TLR4-/- Mice With CDI

**Figure 2:**
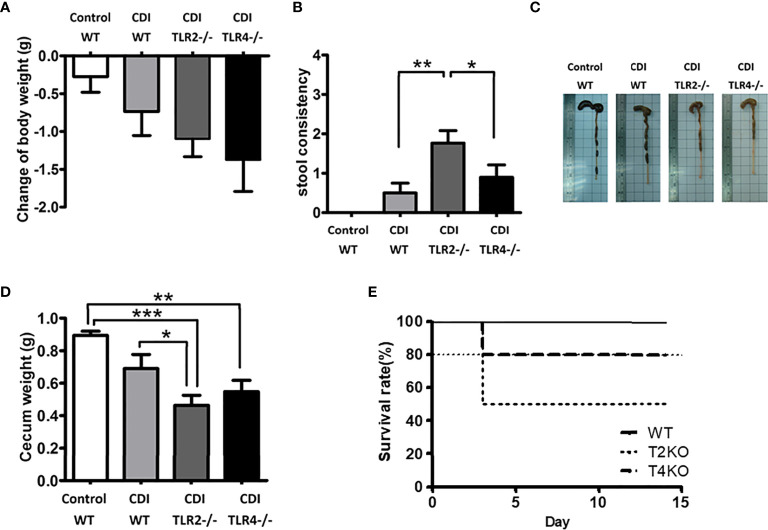
Disease progression of CDI in mice of different genotypic backgrounds (WT, wild type; TLR2 deficiency: Tlr2-/-; TLR4 deficiency: Tlr4-/-). Mice were first treated with an antibiotic mixture for 2 days and then challenged with *C*. *difficile* or saline. Body weight change **(A)**, stool consistency **(B)**, length **(C)** or weight **(D)** of the cecum and colon at day 2 of infection were measured. Two-week survival rates of the 3 groups with CDI are shown (**E**; n = 23-25 mice/group, *P* = 0.86). Values are expressed as means ± SEMs. (*P < 0.05; **P < 0.01; ***P < 0.001).

To assess the influence of TLR2 and TLR4 on CDI pathogenesis, Tlr2-/- and Tlr4-/- mice were used (**Figure 2**). The mean weight change was normalized to the mean weight at day 0. Tlr2-/- and Tlr4-/- mice both showed more severe CDI symptoms than control mice in terms of body weight change (with a 1.10 g decrease in Tlr2-/- mice and a 1.38 g decrease in Tlr4-/- mice, compared to a 0.74 g decrease in wild-type mice, **Figure 2A**) and fecal content (**Figure 2C**) on the 5th day after challenge with *C. difficile*. Furthermore, Tlr2-/- mice revealed a more severe disease pattern than Tlr4-/-, as evidenced by stool consistency (consistency score: 1.76 for Tlr2-/-, 0.69 for Tlr4-/- mice, 0.50 for wild-type mice, **Figure 2B**), cecum weight (0.46 g for Tlr2-/- mice, 0.55 g for Tlr4-/- mice, 0.69 g in wild-type mice, **Figure 2D**), and lower survival rate (50% for Tlr2-/- mice, 80% for Tlr4-/- mice, 100% in wild-type mice; *P* = 0.86) (**Figure 2E**).

#### Increased Colonic Inflammation in Tlr2-/- or Tlr4-/- Mice With CDI

**Figure 3:**
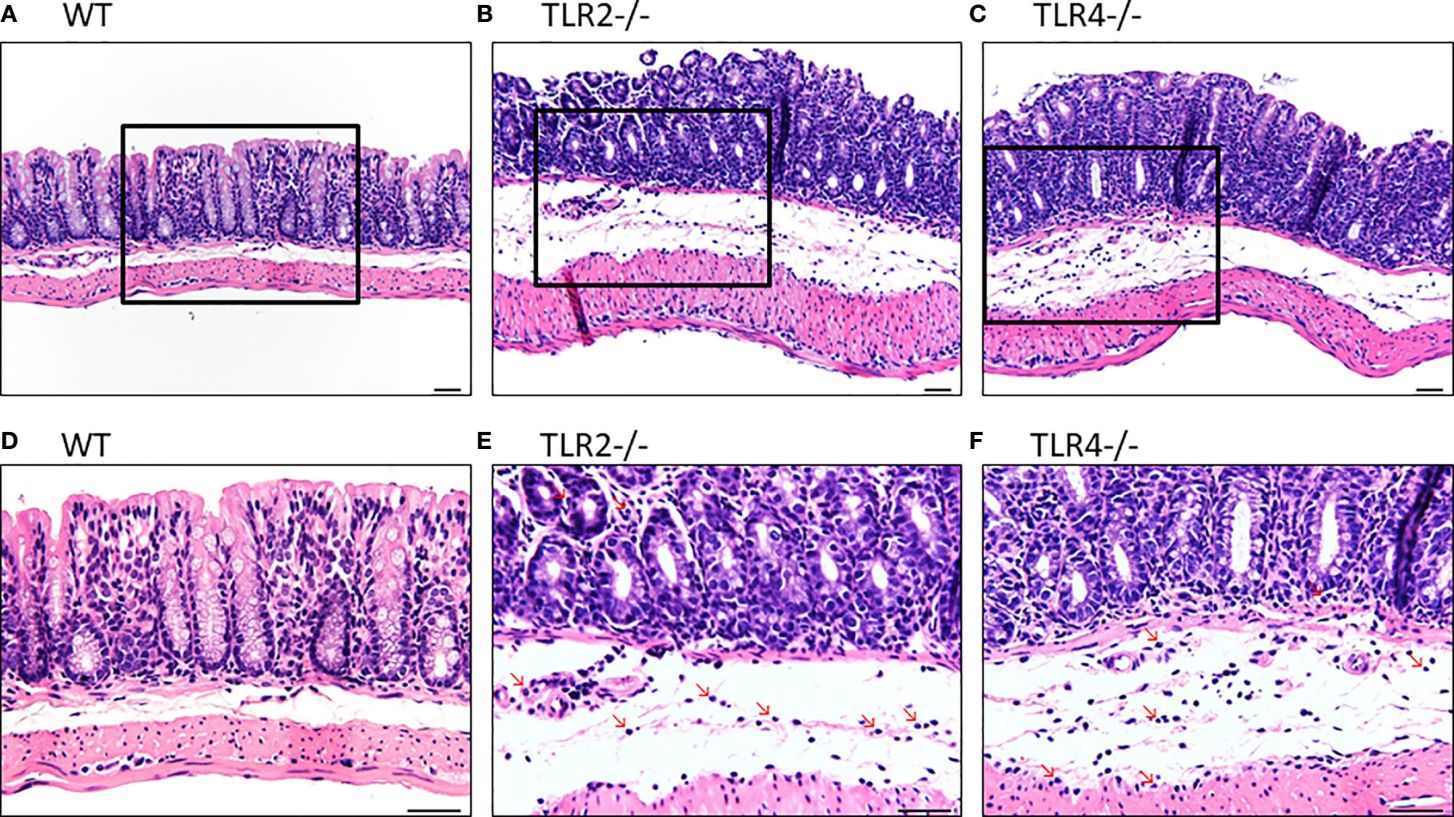
Hematoxylin and eosin staining of colon tissues of mice of different genotypic backgrounds with CDI. Increased infiltration of inflammatory cells, especially neutrophils, in the colonic epithelium was noted in Tlr2-/- **(B, E)** and Tlr4-/- **(C, F)** mice compared with wild-type (WT: **A, D**) mice.

In H&E staining, both Tlr2-/- and Tlr4-/- mice with CDI showed more inflammation than control mice with CDI (**Figure 3**). In addition, Tlr2-/- mice (**Figures 3B, E**) had more inflammation than Tlr4-/- mice (**Figures 3C, F**). Tlr2-/- mice had more neutrophil infiltration (8.0 + 1.6 *vs.* 5.4 + 1.1 and 4.8 + 1.2/HPF; *P* =0.004) but a difference in eosinophil infiltration (1.0 + 0.7 *vs.* 0.4 + 0.5 and 0.2 + 0.4/HPF; *P*=0.08, respectively) than Tlr4-/- mice and wild-type mice. Higher pathologic scores were observed (13.6 ± 1.8 *vs.* 10.0 ± 1.7, and 8.5 ± 1.6; *P =0.001*), especially for neutrophil infiltrates (2.8 ± 0.4 *vs.* 1.6 ± 0.5, and 1.3 ± 0.5; *P*=0.001), mononuclear infiltrates (2.6 ± 0.5 *vs.* 1.8 ± 0.4, and 1.5 ± 0.5, *P*=0.01), and crypt destruction (1.8 ± 0.4 *vs.* 1.2± 0.4, and 1.2± 0.4, *P*=0.06), for Tlr2-/- mice compared to Tlr4-/- mice and wild-type mice. With normalization to each treated genotype, we confirmed that the inflammatory responses were significantly increased in TLR2-deficient mice during CDI, suggesting that TLR2 signaling exerts important immune-protective responses in the intestinal mucosa (**Supplementary Figure 1**).

#### Decreased Colonic Integrity in Tlr2-/- or Tlr4-/- Mice With CDI

**Figure 4:**
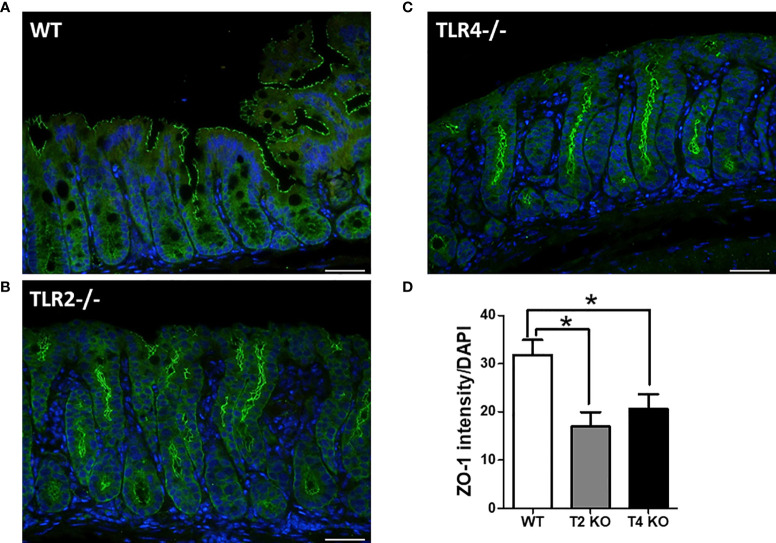
Increased permeability and tight junction protein depletion in colonic epithelium of wild-type (WT, **A**), Tlr2-/- (Tlr2 deficiency; **B**) and Tlr4-/- (Tlr4 deficiency, **C**) mice with CDI, as evidenced by immunofluorescent staining for ZO-1 (green) and Hoechst (blue). ZO-1 depletion was more evident in Tlr2-/- or Tlr4-/- mice than in wild-type (WT) mice **(D)**. Scale bar = 20 μm. (*P < 0.05).

Gut integrity was assessed by measuring the changes in tight junction proteins, especially ZO-1 (**Figure 4**). Less ZO-1 expression (ZO-1 intensity/DAPI) was noted in Tlr2-/- (16.96) and Tlr4-/- (20.56) mice with CDI than in wild-type mice (31.80) with CDI (*P* = 0.02). The original ZO-1 fluorescent staining for each genetic background mouse without infection is illustrated in **Supplementary Figure 2A**. Disseminated aerobic and anaerobic bacteria (**Supplementary Figure 2B**) in the livers of WT and TLR2-deficient mice were also quantified with or without infection. The disseminated bacteria were increased in TLR2 mice even without infection. However, compared to that of WT mice with/without infection, the translocation of bacteria to other sterile organs was increased in TLR2-deficient mice, indicating that intestinal integrity was friable in TLR2 mice, especially during CDI, suggesting that TLR2 might also be involved in the regulation of intestinal permeability during CDI.

## Discussion

In this study, more severe CDI was noted in TLR2- and TLR4-deficient mice than in wild-type mice, and TLR-2 polymorphisms were associated with the occurrence of CDI in the clinic. TLRs, including TLR4 (22) and TLR5 (17), are important host immunity molecules against many bacterial infections and have been proven to play important roles in the pathophysiology of CDI. In this study, we further confirmed the role of TLR2 in host immunity against CDI through clinical and mouse studies. Taken together, TLRs play an important role in innate immunity during CDI. Given the emerging epidemic and decreased efficacy of currently available antimicrobial therapy for severe CDI, immune-based antimicrobial therapy, for example, targeting TLRs, may provide a novel alternative to combat gut infection. A therapeutic strategy targeting TLRs to improve host immunity has been utilized to treat gastrointestinal disease by applying Lactobacillus species; for example, *Lactobacillus crispatus* can modulate epithelial cell defense against *Candida albicans* through the TLR2 and TLR4 pathways (33). *Lactobacillus plantarum*, a commensal bacterium of humans, serves as a regulator of epithelial integrity by activating TLR2 signaling in the gut epithelium (34). TLR expression induced by oral administration of the probiotic *Lactobacillus* modulates cytokine production and improves the immune response against *Salmonella enterica* serovar *Typhimurium* infection in mice (35). Thus, targeting TLR may serve as an alternative therapeutic choice against CDI.

Patients with a TT genotype of the TLR2 rs3804099 (19216T/C) polymorphism were found to be susceptible to CDI in our study. The TLR2 rs3804099 polymorphism has been connected to altered host immunity against bacterial infection (36–38). Of note, in recent years, the TLR2 rs3804099 polymorphism has been thoroughly investigated in association with *Helicobacter pylori* infection, another important pathogen in gastrointestinal disease (37–39). In studies in Iran and in Turkey, patients carrying the TLR2 rs3804099 CT genotype more frequently had peptic ulcers and *H. pylori* infection than healthy individuals (37, 39). Among individuals infected with *H. pylori* in Brazil, harboring the TLR2 rs3804099 (19216T/C) polymorphism had a protective effect in gastric carcinogenesis (38). The linkage of the TLR2 rs3804099 polymorphism to *H. pylori* infection suggests that this polymorphism has an important role in host immunity in the gastrointestinal system, which might explain the association between this polymorphism and CDI in our study.

The pathophysiology of TLR2 in host immunity against CDI was supposed to be correlated with the activation of interleukins because in leprosy patients with the TT genotype, compared to the CC/CT genotype, those with the TLR2 rs3804099 genotype had an increased risk of infection, which might be associated with more than twofold increased expression of IL-6 (40). The peripheral blood leukocytes from trauma patients with the TLR2 rs3804099 CC genotype produce approximately 15-50% greater amounts of IL-10, CXCL8, and TNF-α than those with the TT genotype after bacterial lipoprotein stimulation (24). Additionally, *C. difficile*-derived membrane vesicles can induce the gene expression of proinflammatory cytokines, such as IL-1β, IL-6, and CXCL8, resulting in cytotoxicity in colonic epithelial cells *in vitro* (41). In addition, patients with CDI had higher serum levels of CXCL8 and IL-6 than the normal population (42). In conclusion, according to current evidence, patients with the TT genotype, compared to the CC/CT genotype of the TLR2 rs3804099 polymorphism, might have approximately 15% to more than twofold increased inflammatory cytokine production, which might correlate with disease development during CDI (41).

In this study, TLR4 polymorphism was not associated with the development of CDI; however, in our published study, the TLR4 rs1927914 polymorphism (GG genotype) was associated with *C. difficile* colonization, suggesting that TLR4 polymorphism might lead to adhesion or adaption but does not induce inflammation against *C. difficile* in the gut (23). However, the exact mechanism of TLR2 or TLR4 polymorphisms in the pathogenesis of *C. difficile* colonization or infection warrants further examination.

There are some limitations in our study. First, the mouse model suggested the protective role of TLR2 in CDI. Further investigations of TLR-related innate immunity are needed to clarify the mechanism underlying the role of TLRs in protective immunity against CDI. Second, in the clinical study, the influence of bacterial virulence factors, such as *tcdC* deletion or binary toxins, on the occurrence of CDI was not analyzed. Third, although TLR2 polymorphism was associated with CDI in the univariate analysis, it was not statistically significant in the multivariate analysis, which might be due to the relatively small number of CDI patients in our study; further large-scale study is warranted to clarify the role of TLR2 polymorphisms in CDI. Last, although other TLRs, such as TLR5 or TLR9, were noted to be related to the occurrence of CDI, TLRs other than TLR2 and TLR4 were not analyzed in our clinical and mouse model studies.

In conclusion, TLR2 polymorphism is marginally related to CDI in clinical cases, and TLR2 deficiency is associated with increased disease severity of CDI in a mouse model. The detailed mechanisms warrant further investigation.

## Data Availability Statement

The datasets presented in this study can be found in online repositories. The names of the repository/repositories and accession number(s) can be found in the article/**Supplementary Material**.

## Ethics Statement

The studies involving human participants were reviewed and approved by Institutional review board of Tainan Hospital, Ministry of Health and Welfare, Taiwan-2011014. The patients/participants provided their written informed consent to participate in this study. The animal study was reviewed and approved by Approval of Animal Use: 100143.

## Author Contributions

Y-HL, B-YT, C-YH, Y-HC, P-HC, and Y-LC did the mice model study. H-CL and W-CK did the clinical study. P-JT and Y-PH prepared the article. All authors contributed to the article and approved the submitted version.

## Funding

This study was supported by grants from the Ministry of Health and Welfare (MOHW 106-TDU-B-211-113003) and the Ministry of Science and Technology of Taiwan (MOST 106-2321-B-006-012, 109-2314-B-006-089-MY3 and 108-2320-B-006-043-MY3).

## Conflict of Interest

The authors declare that the research was conducted in the absence of any commercial or financial relationships that could be construed as a potential conflict of interest.

## Supplementary Material

Supplementary Table 1 Probe sequences used for detecting TLR2 and TLR4 polymorphisms.

Supplementary Table 2 Patient characteristics and recent medications of 539 patients with or without the development of CDI. Data are patient numbers (%), unless indicated. iv, intravenous; o, oral.

Supplementary Figure 1 Upon normalization to each treated genotype, the inflammatory responses were significantly increased in TLR2-deficient mice during CDI.

Supplementary Figure 2
**(A)** The original ZO-1 fluorescent staining for each genetic background mouse without infection. **(B)** Disseminated aerobic and anaerobic bacteria in the livers of WT and TLR2-deficient mice were also assessed with or without infection. The disseminated bacteria were increased in TLR2 mice even without infection. Compared to the WT mice with/without infection group, the amount of bacteria translocated to other sterile organs was increased in TLR2-deficient mice, indicating that intestinal integrity was friable in TLR2 mice, especially during CDI.
