# Corrigendum: The Role of Toll-Like Receptor-2 in *Clostridioides difficile* Infection: Evidence From a Mouse Model and Clinical Patients

**DOI:** 10.3389/fimmu.2021.803805

**Published:** 2021-12-08

**Authors:** Yi-Hsin Lai, Bo-Yang Tsai, Chih-Yu Hsu, Yi-Hsuan Chen, Po-Han Chou, Yueh-Lin Chen, Hsiao-Chieh Liu, Wen-Chien Ko, Pei-Jane Tsai, Yuan-Pin Hung

**Affiliations:** ^1^ Institute of Basic Medical Sciences, College of Medicine, National Cheng Kung University, Tainan, Taiwan; ^2^ Department of Medical Laboratory Science and Biotechnology, National Cheng Kung University, Tainan, Taiwan; ^3^ Department of Internal Medicine, National Cheng Kung University Hospital, Tainan, Taiwan; ^4^ Departments of Internal Medicine, Tainan Hospital, Ministry of Health & Welfare, Tainan, Taiwan; ^5^ Department of Medicine, College of Medicine, National Cheng Kung University, Tainan City, Taiwan; ^6^ Center of Infectious Disease and Signaling Research, National Cheng Kung University, Tainan City, Taiwan; ^7^ Department of Pathology, National Cheng Kung University Hospital, Tainan, Taiwan

**Keywords:** *Clostridioides difficile* infection, toll-like receptor, TLR2, rs3804099, tight junction, mouse model

In the published article, there was an error regarding the affiliation for **Yuan-Pin Hung**. As well as having affiliations 3 and 4, they should also have “Department of Medicine, College of Medicine, National Cheng Kung University, Tainan City, Taiwan”.

The authors apologize for this error and state that this does not change the scientific conclusions of the article in any way. The original article has been updated.

## Publisher’s Note

All claims expressed in this article are solely those of the authors and do not necessarily represent those of their affiliated organizations, or those of the publisher, the editors and the reviewers. Any product that may be evaluated in this article, or claim that may be made by its manufacturer, is not guaranteed or endorsed by the publisher.

