# Supplementary material for: The Role of Toll-Like Receptor-2 in Clostridioides difficile Infection: Evidence From a Mouse Model and Clinical Patients
Source: Front Immunol. 2021 Jul 12;12:691039. doi: 10.3389/fimmu.2021.691039 (PMC8313301; doi:10.3389/fimmu.2021.691039)
Supplement: Supplementary Table 1 — Probe sequences used for detecting TLR2 and TLR4 polymorphisms. [file DataSheet_1.docx]

**Supplementary Table 1.** Probe sequences used for detecting TLR2 and TLR4 polymorphisms.

| Gene  (rs number) | Allele | Probes |
| --- | --- | --- |
| TLR2 |  |  |
| rs1898830 | A/G | Probe: 5-ATAGTAAAATAAATCCAGAGAAATC[A/G]GAACAGGGGAAATAATAATATAAGA-3 |
| rs3804099 | C/T | Probe: 5-CAAAAAGTTTGAAGTCAATTCAGAA[C/T]GTAAGTCATCTGATCCTTCATATGA-3 |
| rs7656411 | G/T | Probe: 5-TTTTTAAGCAAATATATACCTAGAG[G/T]TTCCTCATAATGACTCAAAAATAGT-3 |
| TLR4 |  |  |
| rs10983755 | A/G | Probe: 5-TCCCTCACAGCTTGGTTTTTGACAC[A/G]TTGGATTGGAAGTGCTTGGAGGATA-3 |
| rs1927914 | A/G | Probe:5-AGTAGAACTATCTAGGACTTAGCAT[A/G]CATAATATTCCTGTTTTAAATCAGG-3 |

**Supplementary Table 2:** Patient characteristics and recent medications of 539 patients with or without the development of CDI.

| Variables | CDI | | *P* value |
| --- | --- | --- | --- |
|  | No, n=508 | Yes, n=31 |  |
| Male gender | 280 (55.1) | 18 (58.1) | 0.75 |
| Age, mean ± standard deviation (years) | 72.9±15.4 | 72.4±11.3 | 0.86 |
| Nasogastric tube feeding | 250 (49.2) | 20 (64.5) | 0.10 |
| Hospitalization duration (days) | 20.9±16.8 | 36.7±22.5 | <0.001 |
| Underlying disease |  |  |  |
| Hypertension | 256 (50.4) | 11 (35.5) | 0.11 |
| Old stroke | 199 (39.2) | 12 (38.7) | 0.96 |
| Diabetes mellitus | 166 (32.7) | 20 (64.5) | <0.001 |
| Chronic kidney disease | 74 (14.6) | 5 (16.1) | 0.81 |
| Chronic obstructive pulmonary disease | 69 (13.6) | 2 (6.5) | 0.25 |
| Congestive heart failure | 41 (8.1) | 4 (12.9) | 0.32 |
| Malignancy | 35 (6.9) | 4 (12.9) | 0.27 |
| Liver cirrhosis | 7 (1.4) | 0 (0) | 1.00 |
| Cephalosporins | 380 (74.8) | 30 (96.8) | 0.005 |
| Cefazolin, iv | 18 (3.5) | 0 (0) | 0.29 |
| Cefuroxime, iv/o | 53 (10.4) | 1 (3.2) | 0.19 |
| Ceftazidime or ceftriaxone, iv | 305 (60.0) | 26 (83.9) | 0.008 |
| Cefepime, iv | 115 (22.6) | 12 (38.7) | 0.04 |
| Fluoroquinolones, iv/o | 26 (5.1) | 2 (6.5) | 0.67 |
| Penicillins other than piperacillin-tazobactam, iv/o | 102 (20.1) | 2 (6.5) | 0.06 |
| Piperacillin-tazobactam, iv | 79 (15.6) | 6 (19.4) | 0.61 |
| Carbapenem, iv | 147 (28.9) | 13 (41.9) | 0.12 |
| Glycopeptide, iv | 102 (20.1) | 6 (19.4) | 0.92 |
| Metronidazole, iv/o | 18 (3.5) | 0 (0) | 0.61 |
| Fosfomycin, iv | 21 (4.1) | 1 (3.2) | 1.00 |
| Proton pump inhibitors, iv/o | 76 (15.0) | 11 (35.5) | 0.003 |
| H2-receptor antagonists, iv/o | 69 (13.6) | 7 (22.6) | 0.18 |
| Steroid, iv/o | 120 (23.6) | 4 (12.9) | 0.17 |

Data are patient numbers (%), unless indicated.

iv = intravenous; o = oral
