# Supplementary figures and images for: The Role of Toll-Like Receptor-2 in Clostridioides difficile Infection: Evidence From a Mouse Model and Clinical Patients
Source: Front Immunol. 2021 Jul 12;12:691039. doi: 10.3389/fimmu.2021.691039 (PMC8313301; doi:10.3389/fimmu.2021.691039)

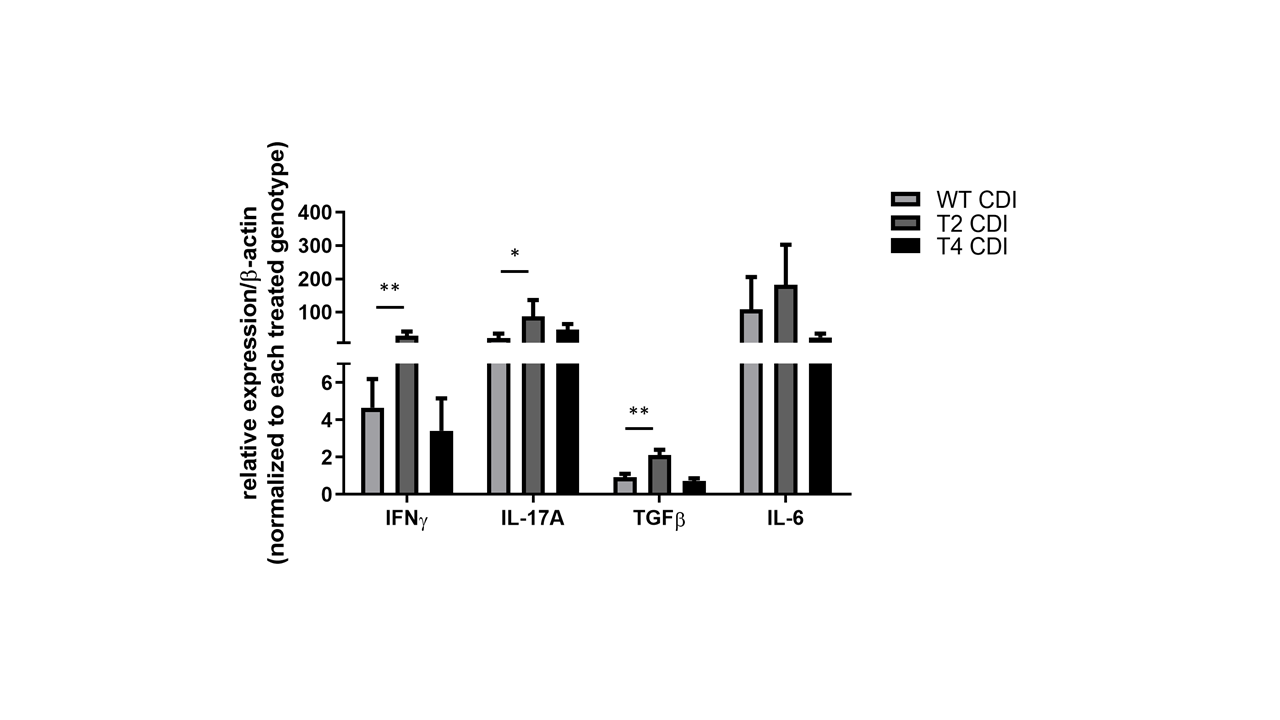

Supplement: Supplementary Figure 1 — Upon normalization to each treated genotype, the inflammatory responses were significantly increased in TLR2-deficient mice during CDI. [file Image_1.tif]

A

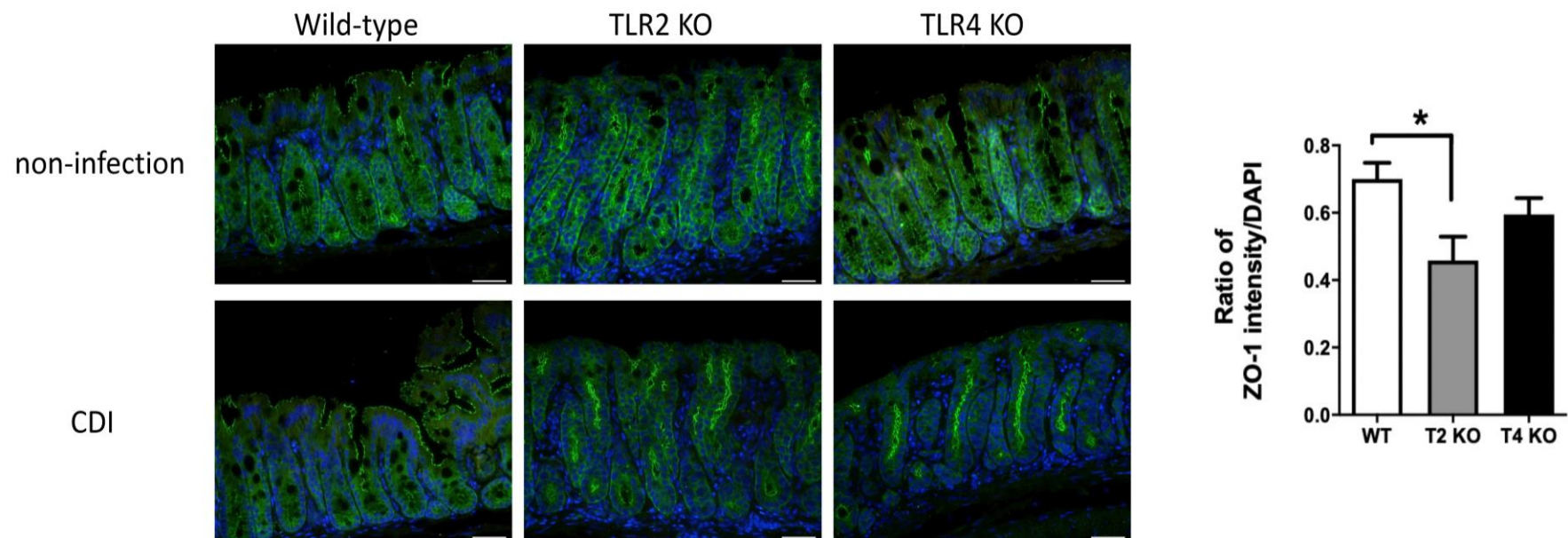

B

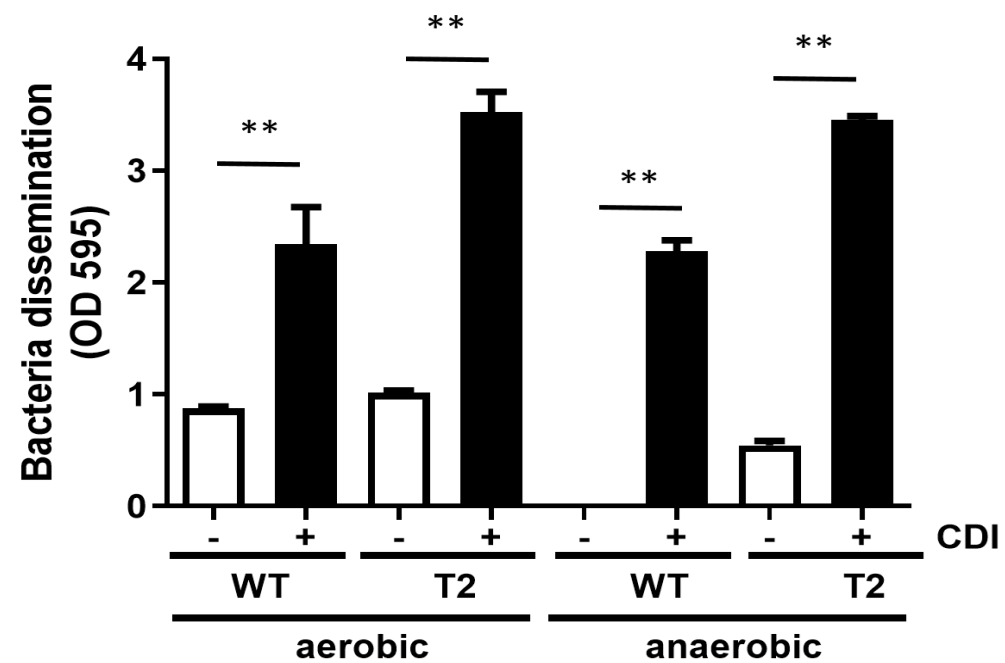

Supplement: Supplementary Figure 2 — (A) The original ZO-1 fluorescent staining for each genetic background mouse without infection. (B) Disseminated aerobic and anaerobic bacteria in the livers of WT and TLR2-deficient mice were also assessed with or without infection. The disseminated bacteria were increased in TLR2 mice even without infection. Compared to the WT mice with/without infection group, the amount of bacteria translocated to other sterile organs was increased in TLR2-deficient mice, indicating that intestinal integrity was friable in TLR2 mice, especially during CDI. [file Image_2.pdf]

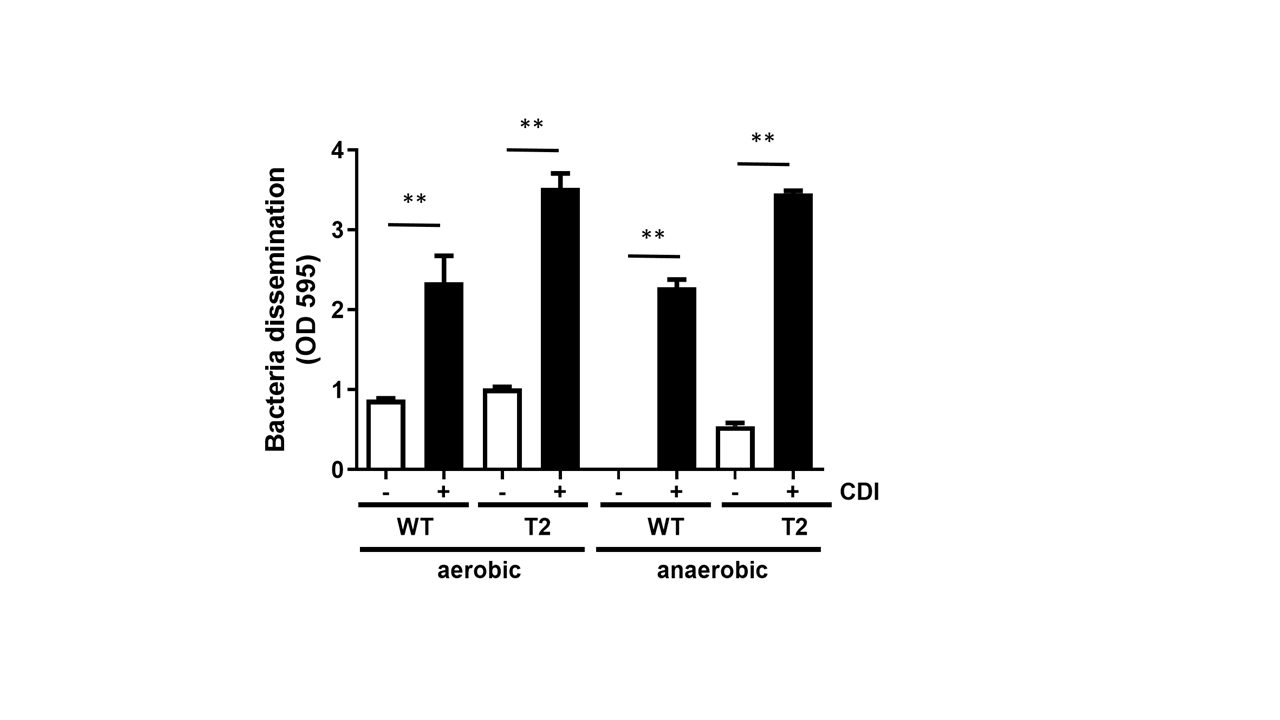

Supplement: Supplementary file 4 [file Image_3.tif]
